# Narrative historical review of scratch-and-sniff books and their key storytelling features

**DOI:** 10.1177/20416695241257566

**Published:** 2024-06-11

**Authors:** Charles Spence, Natalia Kucirkova, Janine Campbell, Yang Gao, Jas Brooks

**Affiliations:** 6396 University of Oxford; 56627 University of Stavanger; 56627 University of Stavanger; 6396 University of Oxford; 189299 University of Chicago

**Keywords:** scent, storytelling, development, books, reading

## Abstract

This conceptual paper examines the use of odours and scents in books to enhance storytelling and engage readers. While books often possess a distinctive smell linked to their material production, the intentional use of scents in books is rare. Our study focuses on scratch-and-sniff books, examining their narrative purposes and contributions to young children's literature. We conduct a narrative historical review, supplemented by a systematic search of databases, online catalogues and lists, to identify a collection of these scented books. Through this review, we explore the extent to which these books represent a unique category of children's picture books, investigating how their features align with theoretical understandings of quality characteristics in children's literature and the role of olfactory cues in storytelling. We address why most scented books target younger readers and discuss possible reasons for the absence of scented books for an adult readership. This intriguing asymmetry contrasts the use of scent in other media (such as film, theatre or virtual reality), often directed toward adults. In addition, this review sheds light on the innovative use of scents in books and their impact on reader immersion and narrative experience. Finally, we consider possible future uses of scent in the context of digital books (ebooks).

Many traditional, paper-based, or physical books have a distinctive smell that reveals something about the materials used in their creation (e.g., Lattuati-Derieux et al., 2006; Strlič et al., 2009). Readers sometimes acquire an emotional or sentimental attraction to the distinctive smell of particular books (Armistead, 2017; Bilton, 2012; McLaughlin, 2015; Spence, 2020b). However, there are far fewer examples where scents and smells have been deliberately introduced into books to augment the story or the readers’ engagement with the narrative. In this conceptual review, we take a closer look at the scent-enhanced books that have been published to date and try to establish the narrative purposes of the scents within some of those books. We use the methodology of a narrative historical review (see Ferrari, 2015; Furley & Goldschmied, 2021, for the strengths of this format), supplemented with a systematic search for odour-enhanced books. We aim to establish the extent to which currently published scratch-and-sniff books represent a unique category of children's picture books and how their key features map onto theoretical notions of what is known about the quality characteristics of children's picture books (e.g., readers’ story immersion). We are also interested in the uses of olfactory stimuli in storytelling (e.g., the pleonastic use of smells in analogue and digital formats; Banes, 2001).

## Children's Picture Books

Picture books, also known as *picturebooks*, convey meaning through both textual and visual or iconic (picture-driven) narrative (Nikolajeva & Scott, 2013). As the compound term indicates, pictures and words are used equally within picturebooks. Typical picture books are “a large picture on each double-page spread, usually accompanied by a short verbal text” (Nodelman, 1996). Picture books are a specific literary genre, which “as an art form hinges on the interdependence of pictures and words, on the simultaneous display of two facing pages, and on the drama of turning the page” (Bader, 1976, p. 1). Picture books are not merely words illustrated by pictures, but rather both pictures and words that complement each other in delivering the meaning of the text and creating tension, or drama, to encourage readers to progress in a narrative.

In the West, the picture book format is primarily associated with children's reading (however, see Sipe, 2008, who argues that the term should apply to readers of any age). Studies of the materiality of picture books (their textures, weight, and other format-related characteristics) have drawn attention to the multisensory nature of many picture books for the youngest children (such as baby books that are often wordless but come with several possibilities for children to touch and manipulate them, see Beauvais, 2023). Online descriptions of these books tend to draw attention to the opportunity for pleasurable shared sensory experiences involving the very young child and the reader.

## Scratch-and-Sniff Technique

Currently, fragrance microencapsulation (or scratch-and-sniff media) is the most widely used technology to embed odours into printed media. This technique comprise paper coated in tiny plastic capsules of fragrance oil. The technique received the colloquial name “scratch-and-sniff” from its fragrance delivery mechanism: by applying pressure to scratch the surface, the microscopic capsules rupture and release their fragrance. Although the microencapsulated fragrance does not necessitate an associated graphic, scratch-and-sniff surfaces typically include graphics and images as additional cues to guide users’ interaction with the surfaces (Brooks & Lopes, 2023).

An early example is Desmond Marwood's *The Enchanted Island* (first published 1971). The book is 28 pages long (of which 26 include the illustrated picture book story) and includes 14 scented pages. The pages containing scratch-and-sniff images have their page number circled. The olfactory stimuli relate to food and drink items, namely fruits, sweets, cakes, juice, and one non-food item, a minty-smelling toothpaste. This limited-release volume was among the first commercial scratch-and-sniff books.

Several issues and limitations exist in the development of scratch-and-sniff picture books, which may have limited the success of such books, including the fact that the scented element is likely to fade over time (and repeated use). This study aimed to identify as many examples as possible where odours were added to picture books and establish how the genre has evolved throughout history since its first publication. We were particularly keen to explore the role of added odours in the book, that is, how they align with current theories on educational design.

## Theoretical Framework

According to cognitive load theory, the capacity of the human memory system is limited and cannot process large amounts of information all at once, but the design of instructional materials and learning environments can optimise processing (Mayer & Moreno, 2003). The need for readers to scratch-and-sniff a scent can be seen both in terms of enhanced engagement with a book but also as breaking the flow of storytelling and the immersion in the story (Winkielman et al., 2003) as the focus switches from the story to the activity of scratching and sniffing (Spence, 2020a). This may increase the cognitive load for readers. This hypothes aligns with Mayer's (2005) multimedia theory and the coherence effect, according to which students learn more deeply when extraneous material is excluded rather than included. Extraneous material, in the case of children's picture books, refers to those elements that are not directly related to the story. According to multimedia theory, adding more elements to the reading experience can be distracting rather than learning-enabling for young readers.

Cognitive load theory is particularly important when considering the “pleonastic” use of odours in picture books. This term refers to the use of smell to match the scent of what is described (or depicted, in the case of an illustration). This approach of olfactorily representing something that is already visually apparent has been criticised by Banes (2001), in the context of scent's use in a live performance setting. Focusing on performances primarily targeted at adult audiences, Banes highlights the redundancy of adding olfactory information to visual and auditory (or, in books, textual) information merely duplicates, rather than expands, meaning. This, in turn, would increase the cognitive load for the viewers or readers.

In this study we set out to systematically examine the various uses of odours in picture books, including their possible pleonastic use. We aimed to connect with existing literature regarding the potential uses of scents in books and their roles and critiques with a systematic review of books that are available on the market, investigating various catalogs and online lists of scratch-and-sniff books. In addition to the systematic search, we compared and supplemented our findings with an open database of children's scratch-and-sniff books (Brooks, 2020). This empirical basis enabled us to gain a deeper understanding of the available resources, thereby allowing us to provide more comprehensive commentary on the added value of odors in children's books.

## Methods

A multi-step search strategy was used to identify and map as close to all scent enhanced books in the English language as possible. All searches were conducted between November 27 and 30, 2023.

First, a search for academic and other publications discussing scratch-and-sniff books, using the search string “(“scratch and sniff” OR “scratch & sniff”) AND book”, was conducted with both Google Scholar (18 items) and EBSCO (covering the libraries of Academic Search Premier + ERIC + SOCindex) (138 items). These items were combined, with duplicates removed, resulting in 106 unique items which, once screened, resulted in just 6 items that mentioned any specific scratch-and-sniff books by title. Since the few books mentioned in these items clearly did not come close to a comprehensive list of published scratch-and-sniff books, we therefore continued with a search of book databases, catalogues, and other lists.

This second stage of searches involved searching lists from popular online booksellers for all English language book titles mentioning scratch-and-sniff. The booksellers were Amazon (636 items), Booktopia (33 items), and Barnes & Noble (47 items). In recording the details of the items on these lists that were, in fact, scratch-and-sniff books, several online lists of books were also identified and reviewed, including “Bookriot: Scratch and sniff books for kids” (15 items), “Bookriot: Scratch and sniff books for grown-ups” (6 items), and Goodreads scratch and sniff list (73 items). Finally, we accessed a published list of scent-enhanced books (Brooks, 2020) that included in-depth information on 130 items.

**Figure 1.**
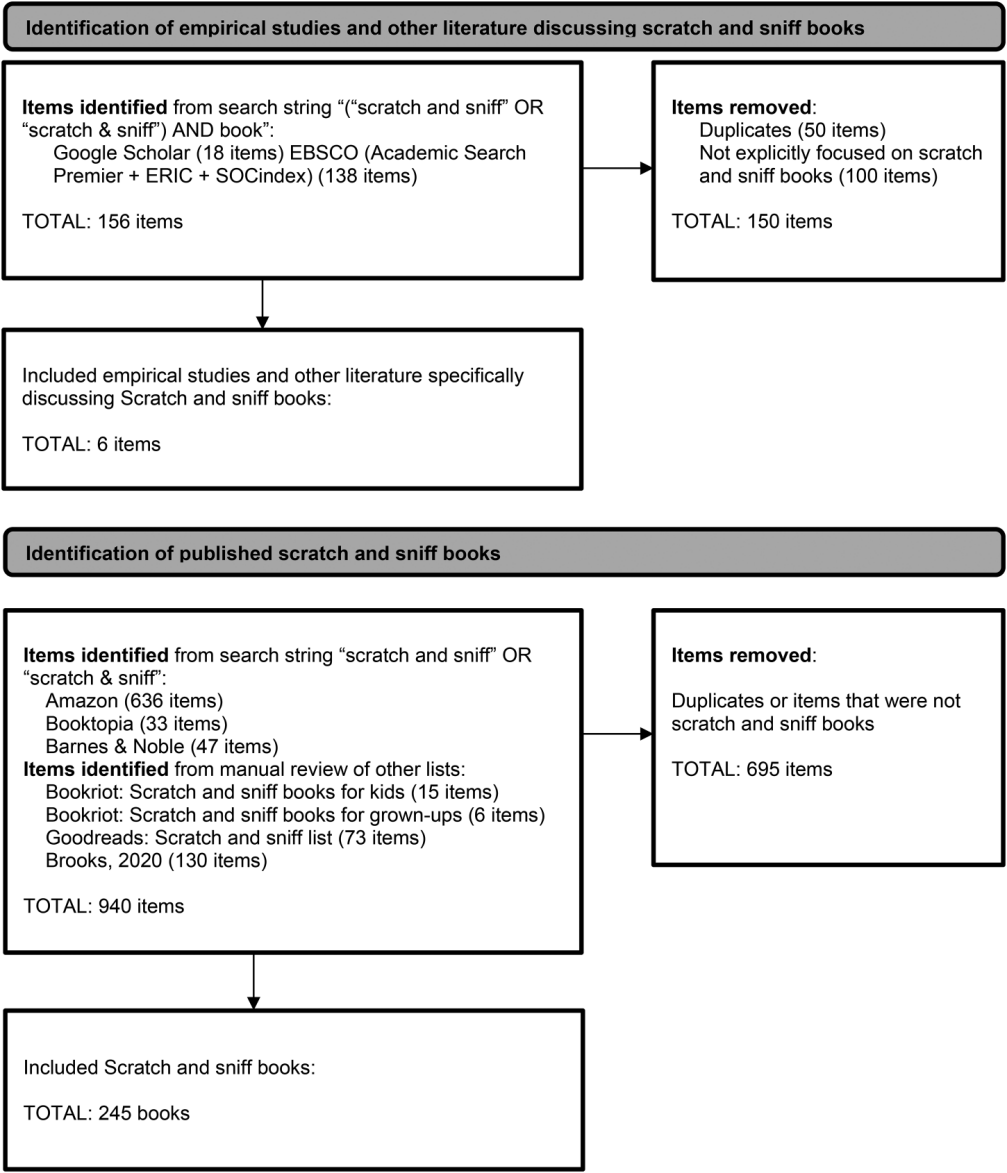
Flow chart of systematic search resulting in 245 scratch-and-sniff books.

The result of this multi-stage search process, illustrated in the flow chart Figure 1, was 245 unique items, each representing a scent enhanced book that was, or still is, available for purchase. The full list of identified books can be found in Supplemental Table 1.

## Results

### Descriptive Findings

**Figure 2.**
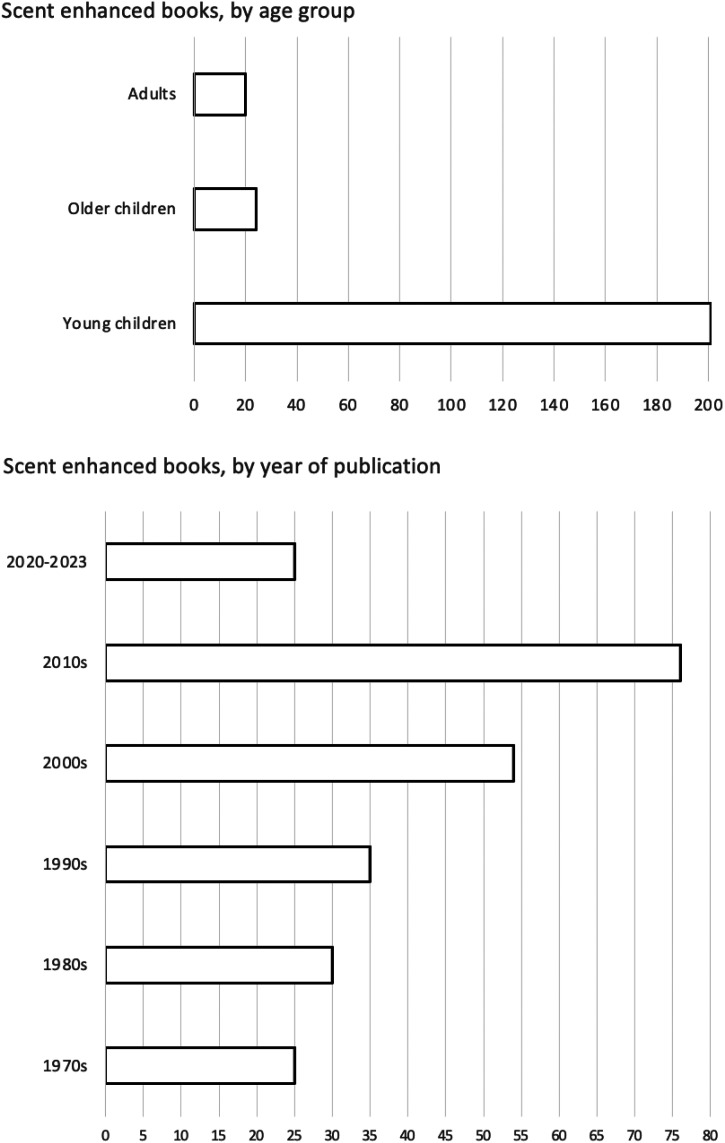
Distribution of scratch-and-sniff books.

The 245 scent-enhanced books identified in this multi-stage review were first analysed using descriptive statistics. As illustrated in Figure 2, the distribution across the age of the intended target audience was heavily skewed towards young children (including target readership up to 8 years, young children, and young readers), with 201 books (82%), with 24 books (10%) developed for children 8 and over, and just 20 books (8%) developed for adults. The distribution of items across publication decades (also included in Figure 2) shows steady growth since the first publications in 1971, with 76 books (31%) published during the decade of the 2010s, and 25 books (10%) already published in the first three years of the 2020s.

In terms of the length of the books and number of scents included, there is, on average, 26 pages per book, but this is lower for books published for children (17 pages for children under 8 years of age, and 33 pages for children 8 years of age and over) than for adults (with the average book length being 73 pages, which is heavily skewed by several very long books, for example, De Cupere, 2017). The average number of scents included in each book showed a similar, although less dramatic pattern across age groups, with 7, 8, and 11 scents on average in each respective age group.

An analysis of the topical content of the books (coded for content by authors, based on the online catalogue descriptions) showed that the largest category was of books focussing primarily on the scents of food, with 112 books (46%), followed by books focussing on the scents of holidays and special days (such as Christmas, Thanksgiving, Halloween, Valentine's Day, Easter, Birthdays) with 50 books (20%). A significant number of books, 47 (19%), were published in the context of popular TV series or extensions of established intellectual property/franchises (such as Star Wars, Sesame Street, Strawberry Shortcake, Harry Potter, Scooby Doo, Smurfs, Hello Kitty, Spongebob, etc.). There was, naturally, some overlap between these categories.

When looking at tokenized scents (e.g., “apple pie” became “apple” and “pie”), we found that malodorous books that focused solely on bad smells (such as the earliest example *The stinky book: A scratch & retch book;* Lukes, 1993) were also relatively common (28 books). This category did not overlap with the previously described categories.

Not all available book descriptions included a list of the scents covered by the scratch-and-sniff content, but from those that did, the 10 most frequent scents were: chocolate, strawberry, peppermint, pine, apple, candy, gingerbread, orange, cinnamon, and pie. From this list, pine is the only non-food scent, and is no doubt related to the frequency of scratch-and-sniff books about Christmas.

### Scratch-and-Sniff Picture Books for Younger Children

The majority of the books identified in this review (*n* = 201, 82%) have been produced for young children (under 8 years old). This is true for every decade, with the numbers steadily increasing across time, from 20 books in the 1970s to 57 books in the 2010s (and already 21 books between 2020 and 2023, including two books announced for publication in 2024).

Of the 11 books identified in this review as being published before 1975, all were targeted at younger readers, with the majority following the storyline of an animal character (dog, bunny, bear, or kitten) following their nose into either adventure (making or finding a birthday cake, exploring a garden, finding something lost) or misadventure (getting into trouble around home or at the marketplace). The adventure format was common across scratch-and-sniff books for young children from all decades.

The publication of multisensory books, including elements of touch and feel, lift and see, scratch and sniff, and even sound, has undoubtedly become more common over the decades. A compelling example is the series of three books (Irwin, 2018) designed specifically for children of all abilities to learn to count, including large print, scratch and sniff, and braille. Other multisensory books focus on a wide range of topics, including nature, Christmas, baking, and even the Moon of Endor (*Star Wars*) (Holt, 1986).

In general, odour-enhanced books targeted at younger readers tend to be shorter than those developed for older readers (on average, 17 pages long) and, accordingly, have fewer scents per book (on average, 7 scents).

### Scratch-and-Sniff Picture Books for Older Children

Only 24 of the books identified in this review (10%) were produced for older children (8 years and older). Nine of these books are from the “Smelly Old History” series (Dobson, 1997, 1998), covering the aromas and stenches of the Romans, Tudors, Victorians, Greeks, Royals, Vikings, and wartime, mummies, and medieval villages. These books are each 32 pages long, with five odours per book.

Other examples of odour-enhanced books for older children include a book of poems (Scholastic, 1975) and a satirical book about farts, featuring what is described as a “great killer fart” (Kavet, 1997). Notably, several scented books targeted at this age group focus on malodours. Many include pungent fragrances (e.g., onion). However, most books recontextualize common fragrances. For instance, Kavet's book uses common scratch-and-sniff fragrances (like fish, cheese, and floral) but labels and contextualizes them as different types of farts. Similarly, *Little Monster's Scratch and Sniff Mystery* uses labelling to alienate the reader from everyday smells. This approach appears in several books focused on unpleasant smells, such as *The truly tasteless scratch and sniff book* (Donkin, 2000) and *Smelly Stories* (Igloo, 2012). Approximately half of the malodorous books identified in this review have been produced for this age group.

### Scratch-and-Sniff Picture Books for Adults

Outside of children's books, there would appear to be little deliberate use of scent in books, with only 20 of the books identified in this review produced with adults in mind. While children's picture books come with the implied readership of adults and children, odour-enhanced books for adults are presumably targeted solely at an adult readership, and the topics of these books reflect this. Adult scratch-and-sniff books include books on whisk(e)y, wine, beer, and cannabis, a series of travel and food books, and two books focused on art (Barry, 2015; De Cupere, 2017).

Examples of odour-enhanced book content for adults include Betts’ guides to wine, whiskey, beer, and cannabis (2013–2021). Each book is 22 pages long, and although not stated for all volumes, the guides to becoming a wine expert and a whiskey know-it-all each include 16 scents. Each book is a fact-filled, scented exploration of these consumables’ basic components, science, and production processes.

Peter De Cupere's *Scent in Context: Olfactory Art* (2017) provides another notable example of odour-enhanced books for adults. The book includes 22 images paired with 11 scents described as smelling like an ashtray, baby lotion, cardamom, cedar, gardenia, grass, horse manure, marijuana, peppermint, strawberry, or woodland. Despite its rich content, the book's substantial size (472 pages) and lack of clear indicators as to the scents’ locations turn the reading experience into something of an olfactory game of hide-and-seek. This challenge raises interesting questions about adult readers’ engagement with such a format. It would be interesting to investigate how readers navigate and interact with such a hide-and-seek challenge, as would gaining an understanding of the artist's vision for how readers are expected to interact with their work.

## Discussion

In this study, we cast a wide net to identify academic research on scratch-and-sniff books alongside published scratch-and-sniff titles from popular book databases. In total, 245 unique scratch-and-sniff books were identified. However, although the initial searches captured 106 unique empirical items discussing research and mentioning “scratch and sniff” and “book” somewhere in the text, screening of these items identified only six items that meaningfully discussed research with scratch-and-sniff books. Four of these items were periodical articles or book reviews, and only two could be identified as academic research (Kaye, 2001; Kucirkova & Jensen, 2023a, 2023b). Nevertheless, our analysis of published scratch-and-sniff books provided insights into the key trends concerning this book genre, which expands current literature in two primary ways. First, it systemically describes the key themes and target readership of these books. Second, it describes the odours in these books and their functionalities, drawing on the cognitive theories.

### Empiricial Expansion to Current Literature on Scratch-and-Sniff Books

Reviewing currently popular scratch-and-sniff books on the bestselling list in the Amazon bookstore, Kucirkova and Tosun (2023) were able to identify three main ways in which odours tend to be incorporated into books: as additional descriptions of foods, plants, and other objects; as a technique to introduce or sustain humour in a story; and as a tool to entertain children during reading. The present historic narrative review expands on these findings with additional themes portrayed in scratch-and-sniff books over time. Namely, the use of odours as a technique to take readers on a journey, either as an adventure trail, a trip or visit to an unknown place, or simply a tour of scents, seems to be the historically most popular way of using smells.

Our analysis also identified that the actual odours used in books over time tend to be similarly valenced in terms of their hedonic quality. Good smells associated with Christmas, chocolate, cookies, and pie dominate in children's scratch-and-sniff picture books. There is a strong focus on categorizing smells as either good or bad, rather than exploring the abstract qualities of smells. However, a few titles diverge from this trend by delving into the abstract nature of smells, such as by using textual prompts or leading questions. For example, asking the readers “What does this smell feel like?” or “What makes you feel Razzle Dazzle Red?” followed by scratching and sniffing a fragrance.

While books popular in the Amazon bestseller list, as reviewed by Kucirkova and Tosun (2023), seem to predominantly use smell as an add-on feature for readers’ engagement, our historical review reveals that many of the books developed for scratch-and-sniff were crafted with the theme of smell as the central narrative or sensorial element. For example, several titles are concerned with finding lost (or hidden) scents or discovering a character's favourite smell. This approach intrinsically weaves the olfactory element into the storyline, making both the story and smells essential for the readers’ meaning-making within the story.

Another expansion contributed through our systematic search is the enhancement of the list compiled by Brooks (2020). We identified over 100 books that were not included in Brooks’ database and also expanded it further with titles published after 2020. Our dataset thus spans across all years, contributing a comprehensive dataset to the field. We did not have access to the physical copies of the books analysed in the study, so did not conduct a firsthand assessment of the scents. Nevertheless, our contribution sheds light on the underutilization of scent as a concept in storytelling. This observation is elaborated upon in the next section, where we discuss recommendations on how odours can be more effectively utilised, emphasising its potential impact on storytelling.

### Theoretical Contributions to Scratch-and-Sniff Literature

Our theoretical inquiry aimed to investigate whether analysing scratch-and-sniff books can provide insights into the utilization of odours within these books and shed light on whether they qualify as picture books according to the genre conventions of children's literature. To address this inquiry, we analysed the diverse applications of odors in the identified scratch-and-sniff books, reflecting on whether their inclusion merely serves as a redundant sensory element or plays a distinct role in the experience. In doing so, we explore theoretical concepts such as olfactory overload and the contrasting impact of unpleasant versus neutral or pleasant odours on reader immersion within the narrative. In addition, uses of olfactory stimulation outside of children's books served as a reference point for our reflections.

A number of commentators have complained in print about the olfactory overload (unwanted, overly strong odors or the accumulation of scents in a space) that sometimes occurs when a multitude of scents are released in large spaces (such as the cinema, dance hall, or occasionally even in the theatre; Spence, 2020a, 2021a). Such olfactory overload is, however, less likely to be an issue with scratch-and-sniff books, where the release of scent is more controlled by the reader. Here, both the vigour of scratching or rubbing, as well as the distance between the reader's nose and the page, can be independently varied to modify the perceived intensity of the scent emanating from a page. The nature of the smells, whether artificial or natural, also plays a role. Capturing complex natural scents, such as the aroma of freshly baked bread, freshly ground coffee, or premium chocolate, in a microencapsulated form has proven challenging. As a result, there is a risk that the scents embedded in scratch-and-sniff panels may detract from the experience when attention becomes drawn to their synthetic qualities or to a perceived mismatch (or incongruence) between the visual content and associated smell.

Studies involving the incorporation of olfaction into virtual reality suggest that only bad smells (as opposed to neutral or pleasant scents) are capable of enhancing the sense of immersion (Spence, 2021c). These findings are echoed in anecdotal reports concerning the use of scent in installations, such as the Jorvik Viking Museum (Spence, 2020c), and in the cinema, where malodourous scents seem to elicit the comments (Spence, 2020a). Hence, the question of whether the scratch-and-sniff action interrupts immersion in the story may turn out to be contingent upon the hedonic quality of the smell. Although many unpleasant scents were represented in the pages of the olfaction-enhanced picture books, one might also consider the possible well-being benefits that might be associated specifically with the use of pleasant scents (see Spence, 2020d).

Critics of scratch-and-sniff technology in cinema complained that the need to look away from the screen to scratch a panel on a card in the darkened theatre detracted from the viewing experience and could potentially break the experience of flow, or immersion, rather than enhancing it (Spence, 2020a). Overall, there is little evidence that the primary goal of providing scratch-and-sniff elements in children's books is enhanced immersion.

In the context of live performances geared towards adult audiences, Banes (2001) has criticized the practice of simply replicating olfactory elements that are visually depicted on stage, deeming such usage of scent as redundant. Similarly, in the context of reading, this redundancy is observed in fictional picture books where the addition of scent detracts from rather than enhances the narrative. However, our analysis highlights that a majority of scratch-and-sniff books, particularly non-fictional ones, are designed for a younger demographic. In these books, the interactive feature of inviting children to touch and smell images serves an instructional purpose. For instance, when exploring topics like fruits and vegetables, the inclusion of olfactory cues aims to reinforce learning experiences.

According to Boerma et al. (2016), reading picture books for comprehension requires mental imagery skills. It might be argued that literally smelling what is shown visually may unduly constrain the rich olfactory landscape that might have been conjured if readers were to use their own olfactory mental imagery. This aligns with observational studies of parents and children reading scratch-and-sniff picture books together: Kucirkova and Jensen (2023a) observed Norwegian families reading the *Peter Follows His Nose* olfactory picture book and found that regardless of the smell in the provided book, the parent and/or child considered fragrance beyond that provided. However, not everyone finds it easy to create an olfactory mental image of what things actually smell like.^1^ There are marked individual differences in people's self-reported ability to conjure vivid olfactory mental images (see Rouby et al., 2009; Stevenson & Case, 2005; Zhou et al., 2022). As such, providing key scents might play an important role for those whose olfactory mental imagery abilities are limited or who may simply not have had the opportunity to smell the objects (e.g., foods) that are presented. For children reading scratch-and-sniff books with their family members or teachers, the adult mediation of the reading experience means that the actual smelling of the book is facilitated by the adult, who might scratch the book instead of the child, or explicitly encourage the child to scratch and sniff (see Kucirkova & Bruheim Jensen, 2023), thus introducing new interaction patterns with new experiences of the materiality of books.

### Future Directions

The rise of digital technologies (such as ebooks and audiobooks) alongside alternative reading formats opens new possibilities for digitally controlled olfactory delivery.^2^ Especially in digital multisensory applications, there are growing opportunities for computer-controlled scent delivery (see Spence et al., 2017), such as for film, theatre, wearable devices, and cars (Kaye, 2001). Unlike the scratch-and-sniff methods of late 20th-century films, which disrupted immersion by requiring the audience to scratch a card when prompted (Spence, 2017), modern digital formats could seamlessly integrate scents while maintaining the flow of reading without interruption. For example, swiping a digital page to trigger scent release could eliminate the need for areas that need to be scratched and break flow (see Henshaw et al., 2018, on the emerging field of olfactory design). Readers could experience the scent of the madeleine dipped in tea from Proust's famous novel (Van Campen, 2014) or the aromas described in *Perfume* (Süskind, 1989) as they progress through their ebook. Such a development necessitates authors and olfactory experience designers to thoughtfully design user-friendly interaction gestures that align with the natural reading process (Li et al., 2023; Niedenthal et al., 2023). Unlike traditional scratch-and-sniff books where scents are localized to—typically—specific pictured objects with clear boundaries, digital smell delivery may afford more flexible and contextually relevant olfactory cues not just as direct narrative elements (diegetic) but also for scene-setting or evoking abstract concepts like emotions (non-diegetic), akin to mood music in cinema (Kaye, 2001; cf. MasterClass, 2021, on auditory diegesis in cinema).

The narrative historical analysis reported here highlights that the olfactory enhancement of the reading experience is not a digital versus analogue question. For instance, the *Dora the Explorer* scratch-and-sniff book used scents almost exclusively for place-setting, though they were spatially anchored to the relevant odorous object (e.g., flowers in a field). Additionally, scents could be strategically used to evoke specific moods or feelings across any reading format. For instance, releasing the smell of fear might make a ghost story more frightening (see Ackerl et al., 2002; Chen et al., 2006). Some years ago, a notable experiment involved sending final reminder bills infused with androstenone, a chemical linked to social behavior, to subtly coerce recipients into paying (see Spence, 2002). This tested whether scents (and social chemosignaling stimuli or pheromones) needed to be perceived consciously to exert their effect on a person's mood, well-being, or perception. Several titles identified in our review exemplify this possibility. *The hardworking honey bee* (Briggs, 2013) uses essential oil scents to soothe and relax children before bed, representing an ethical and thoughtful use of scent to influence well-being. Similarly, *The Happy Book* by Muldrow (1999) aims to cheer children up through multisensory stimulation. However, the implications of employing such sensory manipulation raise ethical considerations. Additionally, individual reactions to specific scents can vary due to past experiences, which might lead to responses that diverge from the author's intended emotional impact.

Alternatively, one might employ scents like lavender in bedtime books to promote relaxation (Kirk-Smith, 2003a, 2003b) or stimulating fragrances like peppermint to maintain alertness (e.g., Instant Aromatherapy Ltd.). Researchers and teachers have recently started to experiment with the use of ambient scent in the classroom to help manage student emotions and facilitate learning (Ma, 2022; Moss et al., 2017; cf. Jafarzadeh et al., 2013). In such cases, scents serve a functional—rather than narrative—purpose, irrespective of format (analogue or digital). There is also scope to release scene-setting scents, as sometimes used to accompany each act in theatre productions (see Spence, 2021b). However, effectively implementing this in a book format, where the narrative develops over several pages or chapters, presents a unique challenge.

The “sensory turn” in literature, as noted in recent years (e.g., Eshelby, 2008; Nuessel, 2018), reflects a growing trend among authors to engage readers through vivid sensory experiences in reading and other cultural domains. For instance, a recent review of a seafood restaurant encapsulates the heightened attention to sensory elements, describing the textures, flavors, and presentation of dishes in exquisite detail. The article *The Sea, The Sea* (Eshelby, 2008) showcases a culinary narrative that mirrors the literary shift, albeit in distinct contexts. In film, where olfaction has already been incorporated in varying ways, other senses (like taste) are being explored. Edible Cinema (Edible Cinema, n.d.), in London, and Fork n’ Film (*Fork n’ Film | Immersive Cinematic Dining Experience*, n.d.), in New York City and Los Angeles, incorporate dining experiences into film, adding gustatory enhancement to the multisensory experience. Similarly, the “Tate Sensorium” at Tate Modern in 2015 showcased collaborations among a scent designer, an audio specialist, a master chocolatier, and human–computer interaction designers to create multisensory artworks (Pursey & Lomas, 2018). With more attempts in different fields and people's increasing interest in multisensory media, it is noteworthy that there ought to be more opportunities nowadays to augment text with relevant olfactory stimuli. However, as these sensory elements become more prevalent, concerns arise about their potential to interfere with the artist or creator's original intentions (see Pursey & Lomas, 2018). To tackle this issue in the future, authors should incorporate scent into their work from the get-go. They might use scent to serve a narrative purpose and enhance the overall storytelling experience. This integration would presumably have to be a deliberate part of the writing process, rather than an “add-on” element introduced after the completion of the story (Velasco et al., 2018).

The emerging literature on crossmodal correspondence involving olfactory stimuli, even amongst children (see Metatla et al., 2019), suggests an opportunity to engage young readers by introducing almost synaesthetic correspondences in picture books. Such correspondences could involve matching colours, visual shapes, or textures with scents. Our review found several examples that illustrate this possibility. For instance, *The smell of a rainbow* (Goldworm, 2021) associates smells with the colours of the rainbow, and *Shiny, touchy, smelly colours* (North, 2006) is a multisensory photographic book encouraging children to explore the feel and smell of colours. However, one might also want to question whether a picture book would necessarily be the best medium for such multisensory interaction or whether instead it would be best achieved in, for example, a game format. Scents and other sensory stimuli have, after all, long been used in Montessori educational practices (Lillard & Else-Quest, 2006). These reflections support Kucirkova and Jensen's (2023b) conclusion that scratch-and-sniff books for children constitute a new book genre distinct from traditional picture books. This genre's properties and potential applications, particularly in digital formats, remain an area ripe for further exploration and study.

### Study Strengths and Limitations

This narrative review provides both an historical and theoretical perspective on scratch-and-sniff books and the purpose of their olfactory features through a systematic search of academic literature and published books. We are confident that within the definition of our search terms and the timing and scope of the study, we have systematically identified the majority of all published scratch-and-sniff books to date. However, some books may be catalogued online without scratch-and-sniff in their identifying labels, and these may not have been identified. Those books identified were registered online during the narrow window of our searches (November 27–30, 2023), and more books will no doubt have been registered since that time. Finally, some books registered during that period may no longer have online records. An example of this is the *SniffN* books series by Van (2018), registered for sale on Amazon on November 28 (and noted for being a curious mix of scratch-and-sniff for young children and dogs), but no longer able to be found online. Such changes are the nature of searches, no matter how systematic, that are conducted with online catalogues and other less formal lists.

This study's methodology included narrative description of the identified of books, based on the online descriptions of those books. This could helpfully be extended with an in-depth review of each book, more thorough classifications of the characteristics of those texts, and a statistical comparison of those categories. Additionally, future work could include first hand assessments of smells, which could uncover relationships between smell quality and context for pleasant smells akin to how malodour-focused books relabeled common scents to seem bad. (For example, vague odors may benefit from contextual specificity, like a fruity scent being labelled “apple,” or a scent may be both pleonastic and support the mood, such as a dewey rose or a suffocating heavy rose odor depending on the tone of the scene.) These aspects were, however, outside the scope of the current study. Therefore, we recommend that our qualitative review is expanded with a more quantitatively oriented analysis, with the use of appropriate statistical techniques, alongside smell assessments for more robust conclusions.

The limitations we note in the literature are related to the purpose of odour inclusion in books and the technological possibilities for augmenting story experiences with odours. First, a significant critique of incorporating odors in reading experiences is the potential to limit the reader's imagination. Moreover, current technology and affordable scents often smell artificial and synthetic (Spence, 2017), thus potentially failing to enriching the reader's multisensory engagement. As a result, there remains skepticism among the public regarding the value of enhancing their digital reading encounters with scent additions.

Second, the rise of digital reading and e-books potentially opens the way to the augmentation of reading not only by scent but also by sound (e.g., see Ziv & Goshen, 2006) and other interactive elements such as hotspots or video zoom-in features (Bus et al., 2020). However, the inclusion of various add-ons for children's books should not be solely driven by technological possibilities. Instead, it should be guided by carefully considered pedagogical principles and theoretically grounded assumptions about their evidence of positive impact on children's reading experiences. Currently, even with latest advances in smell teleportation such as that promoted by Osmo AI, technology falls short of fully realizing the promise of “the Feelies” described in Huxley's *Barve New World* (1932), which envisaged a form of motion pictures that not only include sight and sound but also tactile and even olfactory sensations (see Frost, 2006). At present, the sonic augmentation of reading may be cheaper and more practical to implement than olfactory augmentation. The cost associated with buying scent technologies and their fragrances is currently prohibitive. Moreover, one of the challenges when working with olfactory augmentation is that it can be difficult to convince people of the beneficial effect of scent. This has been referred to as the fundamental misattribution error, in which people typically attribute their experience, such as pleasure, to one of the other senses instead of smell (Spence et al., 2017). Such considerations, therefore, raise the question of what exactly is special about smell and what experiences or effects can only be achieved through olfactory means, or else be achieved more effectively through smell than sound, say.

In conclusion, most of the examples that have been published to date have been targeted at children. This may help to explain the primarily pleonastic use of scent in such a context (see Banes, 2001). Indeed, given such a context, the incorporation often appears to have more of an experiential, exposure, and/or learning-based focus (Kucirkova, 2022) than any of the more intriguing (and less obvious or direct) ways in which scent can be used in entertainment and narrative contexts (see Banes, 2001). Future research could investigate how incorporating smells into children's books compares to, and perhaps augments, the integration of other interactive elements, such as textures and additional add-on features like “lift-and-see” or “touch-and-feel” components.

Overall, our study contributes to the exploration of why scratch-and-sniff books are predominantly targeted at younger readers despite the potential for olfactory augmentation in storytelling being more commonly directed towards adults in other media. It highlights the asymmetry in the age of the audience and critiques the pleonastic use of scent in books, which might disrupt the immersion in the story. It identifies various examples in children's published books, proposing possible reasons for the absence of scented books for adult readership and discussing potential future applications of scent in digital books.

## Supplemental Material

sj-docx-1-ipe-10.1177_20416695241257566 - Supplemental material for Narrative historical review of scratch-and-sniff books and their key storytelling features Supplemental material, sj-docx-1-ipe-10.1177_20416695241257566 for Narrative historical review of scratch-and-sniff books and their key storytelling features by Charles Spence, Natalia Kucirkova, Janine Campbell, Yang Gao and Jas Brooks in i-Perception

## Notes

1. Even though reading olfactorily redolent words such as “cinnamon” has been shown to lead to the activation of olfactory brain regions (González et al., 2006).

2. That said, it has been widely reported that sales of ebooks appear to have plateaued, and may even be in decline (e.g., Cain, 2017; Spence, 2020b).
