# Supplementary material for: Narrative historical review of scratch-and-sniff books and their key storytelling features
Source: Iperception. 2024 Jun 11;15(3):20416695241257566. doi: 10.1177/20416695241257566 (PMC11168057; doi:10.1177/20416695241257566)
Supplement: sj-docx-1-ipe-10.1177_20416695241257566 - Supplemental material for Narrative historical review of scratch-and-sniff books and their key storytelling features [file sj-docx-1-ipe-10.1177_20416695241257566.docx]

**Appendix A**

Table 1. Comprehensive list of all scratch and sniff books that were identified from

1970 to 2024 and is offered as an open database for academic use:

<https://docs.google.com/spreadsheets/d/1hNKBtIswFOBnynxKgvQEBu1YX31vPqUf/edit?usp=sharing&ouid=109798566887113109135&rtpof=true&sd=true>
